# Malaria Preventive Practices among People Residing in Different Malaria-Endemic Settings in a Township of Myanmar: A Mixed-Methods Study

**DOI:** 10.3390/tropicalmed7110353

**Published:** 2022-11-04

**Authors:** Pyae Linn Aung, Kyawt Mon Win, Tepanata Pumpaibool

**Affiliations:** 1 College of Public Health Sciences, Chulalongkorn University, Bangkok 10330, Thailand; 2 Myanmar Health Network Organization, Yangon 11091, Myanmar; 3 Department of Public Health, Ministry of Health, NayPyiTaw 15011, Myanmar

**Keywords:** endemicity, malaria, mixed methods, Myanmar, preventive practice, rural population

## Abstract

Different villages in a township or an area exhibit different malaria endemicities. This study aimed to investigate malaria preventive practices among people residing in different villages with stratified malaria endemicity in a township in Myanmar. Quantitative data were collected using standardized questionnaires by conducting face-to-face interviews, and qualitative data were obtained through in-depth interviews using a guideline. Quantitative data were analyzed using descriptive statistics and logistic regression models, whereas thematic analysis was used to visualize qualitative data. A total of 360 respondents with similar characteristics participated in the quantitative study. Knowledge regarding malaria and the availability of health information exhibited insignificant differences. Malaria preventive practices were considerably poor among people from moderate- and high-endemic villages (*p* < 0.05). Adult male farmers or gold miners with low annual family incomes reported poor preventive practices in both moderate- and high-endemic groups. People could not practice some malaria preventive measures because of a lack of resources, e.g., a lack of mosquito repellents. To eliminate the disparities of preventive practices in different malaria-endemic settings, heath-awareness-raising activities should be increased, especially in moderate- and high-endemic villages.

## 1. Introduction

Globally, malaria burden has been decreasing each year, but the trend has stalled recently. In 2020, mobility and mortality remained high in Myanmar, with 58,825 malaria cases and 10 malaria-related deaths reported [1]. The current reporting systems of malaria surveillance from villages up to the central level are handled through a password-protected web-based reporting system, in addition to paper-based patient registers managed by data persons at the township, district, and central levels [2]. Village-level micro-stratifications are not yet feasible in Myanmar due to limitations of human resources, data, and budget. Although the national malaria control program (NMCP) is endeavoring to proceed with village-level micro-stratifications, presently, only compiled data at the village tract level have been used as the lowest unit of reporting. However, even in a particular township or village tract with an overall high number of malaria cases, many villages may have fewer malaria cases, or even none at all, despite being in similar geographic locations and having equivalent environmental indicators (such as rainfall) with adjacent villages. Therefore, malaria control strategies and related activities sometimes seem disconnected from the actual situation across each township or area. 

In Myanmar, limited entomologic surveys have found that *Anopheles minimus* and *An. dirus* are the major malaria vectors distributed throughout the country [3]. Insecticide resistance (deltamethrin and permethrin) among these major species remains unreported [4]. Several studies have proven that efficacious malaria preventive behaviors would reduce disease transmission [5,6,7]. The use of long-lasting insecticide-treated nets (LLINs) is internationally recognized as the most effective tool to prevent malaria [8,9]. In Myanmar, insecticide impregnation of conventional nets is implemented only during a malaria outbreak. Otherwise, based on the overall township malaria data, the mass distribution of LLINs is conducted every other year throughout each malaria-endemic township, followed by yearly topping-up replenishments targeting vulnerable malaria groups such as pregnant women, children, and migrant workers. A total of 11,046,132 and 569,016 LLINs were delivered in 2019 and 2020, respectively [1]. Subsequently, LLIN ownership status might be high in targeted high-malaria-prevalence townships. LLIN ownership in at-risk populations across the country overall has increased yearly, from 1.8% in 2011 to 34.3% in 2019 [1]. However, the studies report low use levels of LLINs across different populations: 46.6% among pregnant women [10], 19.5% among children < 5 [11], 52% among migrants [12], and 15.6% in the general population [13]. Therefore, barriers to the proper use of LLINs in the community should be explored. 

Additionally, certain circumstances can prevent people from being able to strictly follow malaria prevention guidelines, namely, forest-related occupations, e.g., rubber tapping or gold mining, which do not allow them to use bed nets because they usually work overnight [14]. In this case, alternative interventions such as wearing long-sleeved shirts/pants and applying mosquito repellents should be promoted. Further, erroneous beliefs about LLINs have been reported, such as that they can be harmful to the skin [15]; along with erroneous beliefs pertaining to alternative means of preventing malaria transmission, such as burning rubbish to prevent mosquito bites, avoiding drinking stagnant water, and eating bananas or papaya to prevent malaria transmission [16]. Therefore, finding the barriers hampering the use of appropriate malaria preventive practices among the community should be conducted. Such information is necessary to formulate specific health promotions or behavioral change strategies to be rolled out, especially when the country is aiming to be malaria-free by 2030.

People living in different locations might have varying levels of malaria preventive knowledge although they practice ongoing similar malaria control activities inside and have equal access to the interventions, including the ownership of insecticidal nets. When the data were reported at the village-tract level, the villages inside each village tract likely received similar control activities. Nevertheless, people’s behaviors toward the use of these measures might differ by locations. Subsequently, this study explored the difference in malaria preventive practices among people residing in different malaria-endemic areas using a quantitative approach and addressed the details of their challenges in following malaria preventive measures using a qualitative approach in a township of the Sagaing Region of Myanmar. The study results would be helpful in formulating new health promotion strategies using separate approaches representing all kinds of malaria-endemic settings. The strategies would be especially applicable in a border area with different malaria endemicities on each side, such as the Thailand–Myanmar border where Thailand struggles to prevent local transmissions due to the many imposing imported malaria cases from Myanmar [17]. 

## 2. Materials and Methods

### 2.1. Study Area

**Figure 1:**
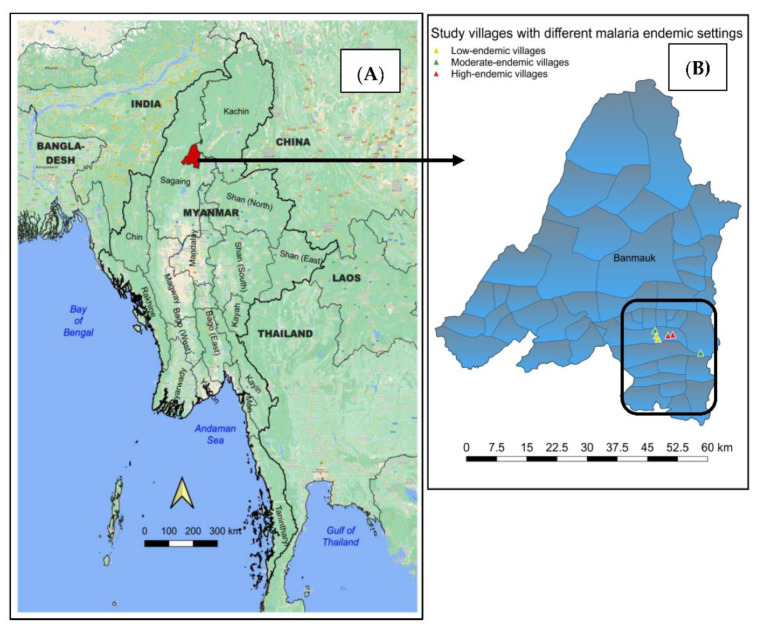
Locations of study township and villages. (**A**) Banmauk Township from the Sagaing Region of Northern Myanmar. (**B**) Six study villages with different malaria situations in a village tract from the township.

The Banmauk Township of the Sagaing Region in Northern Myanmar (Figure 1) was purposely selected as the study area. The majority are working agriculture-related jobs followed by gold mining in some areas. The township has shown a fluctuation of malaria trends in each part of the township as well as inside each village tract. The township-wide annual parasite incidence (API) was 3.8 per 1000 population at risk in 2018. In this township, malaria control activities including monitoring malaria cases using active and passive case detections, treating confirmed cases, and distributing LLINs have been implemented by the township vector-borne disease control (VBDC) team and other malaria partners. One related study in this area [18] concluded that older males were more likely to contract malaria than other individuals. 

A total of 49 village tracts are located in the township. Each village tract typically comprises 10 to 12 villages and an average population of 2000. General well-being, including malaria diagnosis and treatment, is overseen by a government rural health center or subcenter in each village tract. A trained village health volunteer is also assigned to each village by the township VBDC team. 

The most updated available data were 2019 annual data. Therefore, the village tract level data of 2019 were requested from the township VBDC team. Then, the village tracts were ordered by the number of reported malaria cases from the highest to lowest. The top 10 villages with the highest malaria caseloads were listed, and a village tract was then chosen at random from the list.

From the selected village tract, the villages were classified as low-, moderate-, or high-endemic status, based on the 2019 malaria incidence data. The respected APIs of each village from the selected village tract were calculated as the total reported numbers of patients with malaria divided by the per 1000 population at risk. Then, two villages of each endemic setting were chosen using random lottery-style selection for a total of six villages (two villages of low-endemic settings (API < 1); two villages of moderate-endemic settings (API: 1–5), and two villages of high-endemic settings (API > 5)). 

Each village comprised an average of 400 to 600 people and 100 to 150 total households. Currently, the VBDC team is capable of stratifying the malaria stratums only at the village tract level, despite case-based surveillance data being recorded village by village. Therefore, all malaria-related activities were similarly implemented in all villages under a village tract, regardless of the village-wide data. For instance, when the overall village tract’s API is high, only malaria transmission reduction activities are conducted in all villages under that village tract. The VBDC team organized a mass distribution of LLINs during 2019 in all selected villages with a quota of one insecticidal net per 1.8 people. Consequently, all the selected study villages possessed a similar volume of population and malaria control activities, including the frequencies of health-awareness-raising campaigns, the distribution of behavioral change communication materials such as pamphlets and posters created by the national program, and the LLIN distribution strategy.

### 2.2. Study Subjects

The study employed a mixed-methods approach. In late 2021, quantitative research was conducted among representative subjects totaling 360 respondents (120 from each endemic setting), covering about 40% of the total population, or 70% of the households in selected villages. The heads of households or alternative representatives aged 18 years or older, residing in the study villages for two years or more, were eligible to participate in the study. A household listing along with a population census was requested from the respective village authorities, and a systematic random sampling method was applied until the desired sample size was achieved. 

The qualitative assessment focused on participants receiving the lowest total scores for malaria preventive practices during the quantitative evaluation. A total of 20 respondents (an average of seven participants from each cohort) were recruited. Similarities in age, sex, education, and occupation were also considered to enhance the comparability. 

### 2.3. Data Collection

A structured questionnaire with predefined choices in Burmese was used to collect quantitative data. The questionnaire was firstly prepared in English, referencing relevant guidelines [19] and related studies [12,14,20]. It also sought an external review by two malaria experts to ensure its validity. Next, a pretest was conducted in another village tract to test the reliability. Cronbach’s alpha was 0.85. The contents were structured in sequences of the general characteristics of respondents, the assessment of malaria preventive knowledge and practices through predefined choices under the topics of causation of malaria, preventive methods of malaria, basic knowledge on LLIN use, the benefits of using LLINs, and the use of LLINs or other personal protective measures. 

Face-to-face interviews following COVID-19 precautions were held with the help of three data collection assistants with bachelor’s degrees. Each assistant had completed a one-day training course provided by the researchers. The training covered the basic concepts of ethics guidelines, informing of the study objectives and consenting process, the respondent’s rights during an interview, etc. Usually, interview sessions did not last over 15 min. 

Qualitative data were collected through in-depth interviews (IDIs) with the help of the same data assistants. The qualitative guidelines consisted of general characteristics and three open ended questions addressing respondents’ overall insights towards the malaria situation and preventive methods, the challenges faced by respondents in adhering to malaria preventive measures including LLIN use, and the perceived possible solutions to overcoming the obstacles. The interviews took about 30 min. When an interviewer asked a question, another interviewer noted down the respondent’s answers in Burmese. A voice recording was also established during each interview to back up the details of the respondents’ answers.

### 2.4. Data Analysis

The quantitative analyses included five steps. First, respondents’ general characteristics and socioeconomic statuses in each malaria-endemic setting were compared using the numbers, percentages, and a chi-squared test to determine the similarities or to detect any possible bias. Second, the scores for each knowledge question, access to health information, and practice items attained by each group were presented in descriptive styles and categorized the groups based on their “Yes” or “No” answers. Then, the differences among the groups were calculated using chi-squared tests for each statement or question. Third, total practice scores in each group were classified as good (>60% of total scores) or poor (≤60% of total scores) categories, and the scores were illustrated in each group with box plot charts. Fourth, chi-squared tests were used to explore any significant difference between the knowledge categories of each malaria-endemic setting. Lastly, simple and multiple logistic regression models were applied to determine the underlying factors for the poor level of practice scores among people residing in moderate- or high-malaria-endemic villages. All of the constructed variables were included in the adjusted model analysis regardless of their significances during the unadjusted models. Finally, the crude odds ratio and adjusted odds ratio were presented with 95% confidence intervals. All the statistical tests were calculated using the Statistical Package for the Social Sciences (IBM SPSS Statistics for Macintosh, Version 28, IBM Corp., Armonk, NY, USA).

For the qualitative component, the IDI records were first translated to English sentences independently by the researchers. The consistency and correctness were cross-checked and validated using the back-translation method. Then, the transcripts were visualized via thematic analysis with the help of Mindjet-Mind Manager Software (Version 12) [21]. The finalized themes, along with respective quotes, were discussed and agreed upon among by all researchers. 

### 2.5. Ethics Approval

The study protocol was approved by the Institutional Review Board, University of Public Health, Ministry of Health, Yangon, Myanmar (UPH-IRB 2018/Research/29). A signed consent form was also requested from each respondent before collecting the quantitative and qualitative study data.

## 3. Results

### 3.1. Findings from the Quantitative Component

#### 3.1.1. General Characteristics of the Respondents

A total of 360 respondents (120 from low-, moderate-, and high-malaria-endemic settings) participated in this study. Of these, many were >30 to 45 years of age and farmers from families of three to five members with an annual family income of 1,000,000 to 2,000,000 Myanmar Kyats (MMK), and they had obtained primary-school-level education. About one half of the total respondents were males. Using the chi-squared test, all the general characteristics constructed in this study showed insignificant differences in each group (Table 1).

#### 3.1.2. Knowledge on the Causation of Malaria and Access to Malaria-Related Health Information

Respondents from each malaria-endemic setting were asked about knowledge concerning the causation of malaria and the availability of malaria-related health information within their range. Overall, the scores for each knowledge item were similar in each group, except some reported misconceptions among moderate- and high-endemic villages (*p* < 0.05). Respondents from each endemic setting equally accessed malaria-related health information. The source of information was primarily healthcare providers and village malaria volunteers. Still, respondents received that information at an average of only once or twice yearly. Moreover, only about 60% of respondents from each setting acknowledged receiving malaria-prevention-related information through healthcare providers or village health volunteers (Table 2).

#### 3.1.3. Malaria Preventive Practices among Respondents from Each Malaria-Endemic Setting

Respondents from each malaria-endemic setting were assessed regarding detailed malaria preventive practices (Table 3). A high percentage (105, 87.5%) of respondents from low-endemic settings used LLINs, while only one half of the respondents from moderate- (76, 63.3%) and high-endemic settings (67, 55.8%) described the use of LLINs. Still, respondents from each group described that they were using conventional bed nets, following misconceptions such as avoiding eating some fruits and drinking stagnant water to prevent malaria. Only a few respondents in all groups revealed the use of personal protective measures. Some still practiced burning rubbish and using mosquito coils as measures of preventing malaria.

The ownership of LLINs was high among the study respondents in each malaria-endemic setting (>90%). However, many respondents from the low-endemic group (104, 86.7%) used LLINs the night before the survey, while only around one half of the respondents from moderate- (71, 59.2%) and high-endemic settings (60, 50.0%) slept under LLINs the night before the survey. Next, almost all LLINs were likely to be distributed within the acceptable timeframe, i.e., less than two years. Nonetheless, some respondents had already washed the LLINs more than 20 times (Table 3).

#### 3.1.4. Overall Malaria Preventive Practices in Each Malaria-Endemic Setting

**Figure 2:**
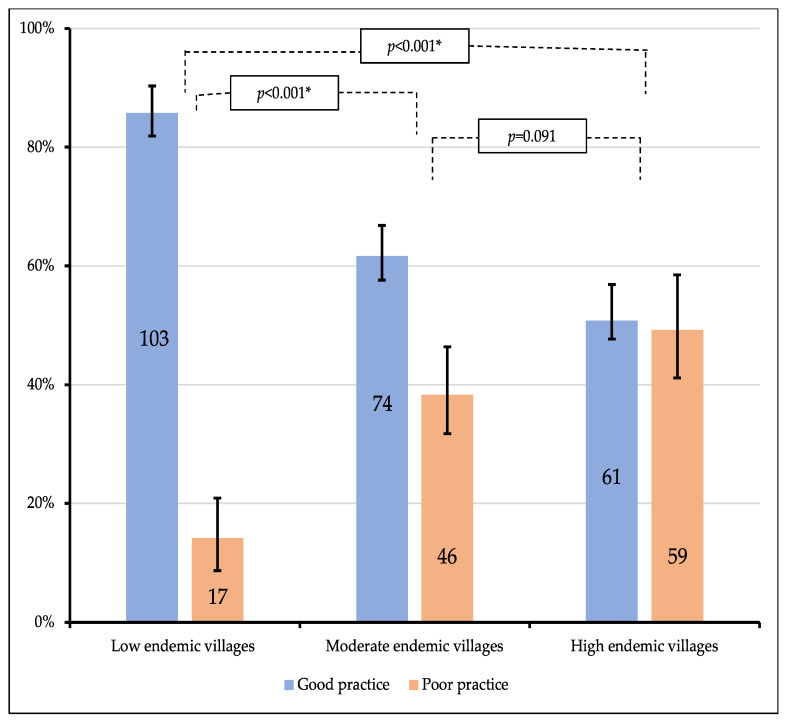
Malaria preventive practices among people residing in low-, moderate-, and high-malaria-endemic villages. (*p*-value by chi-squared test; * statistical significance at *p* < 0.05; degree of freedom = 1).

The practice scores were summarized and categorized as being good or poor. In the low-malaria-endemic setting, more than 85% of the respondents exhibited good preventive practices. However, only 61.7% and 50.8% of the study respondents showed good preventive practices concerning moderate- and high-endemic settings, respectively. The overall scores were compared, and malaria preventive practices significantly differed between the low- and moderate-endemic settings (*p* < 0.001) and the low and high settings (*p* < 0.001). Nevertheless, no statistically significant differences were observed between the moderate- and high-malaria-endemic settings (*p* = 0.091) (Figure 2).

#### 3.1.5. Factors Associated with Poor Malaria Preventive Practices among People Residing in Low-, Moderate-, and High-Malaria-Endemic Villages

In low-malaria-endemic villages, no significant associations were found between the socio-demographics and poor malaria preventive practices (Table S1). 

In moderate-malaria-endemic villages, young adult groups (18 to 45 years) showed higher odds of poor malaria preventive practices than those of older age. The adjusted odds ratio (aOR) and 95% confidence intervals (CI) were 3.1, 1.3 to 8.6 for the age group 18 to 30 years and 3.4, 1.5 to 9.1 for the age group >30 to 45 years, respectively. Males (aOR: 3.1, 95%CI: 2.0 to 8.2) had a higher chance of exhibiting poor preventive practices than females. Concerning type of occupation, farmers (aOR: 4.1, 95%CI: 1.0 to 16.1) and gold miners (aOR: 4.2, 95%CI: 1.5 to 19.5) were more likely to possess poor practices. Additionally, families with low annual incomes (<MMK 1,000,000 (aOR: 4.7, 95%CI: 1.7 to 20.2) and MMK 1,000,000 to 2,000,000 (aOR: 4.1, 95%CI: 1.6 to 14.7)) revealed more poor preventive practices than those with high annual family incomes.

Similarly, males (aOR: 3.6, 95%CI: 2.0 to 9.7), adults (18 to 30 years (aOR: 2.9, 95%CI: 1.4 to 8.1), those >30 to 45 years (aOR:2.8, 95%CI: 1.6 to 11.0)), farmers (aOR: 3.5, 95%CI: 1.9 to 11.2), gold miners (aOR: 2.9, 95%CI: 1.6 to 10.0), and those with an annual family income <MMK 1,000,000 (aOR: 5.0, 95%CI: 2.0 to 17.5) and an annual family income of MMK 1,000,000 to 2,000,000 (aOR: 3.9, 95%CI: 2.1 to 11.3) had underlying risk factors for having poor malaria preventive practices among respondents from high-malaria-endemic villages (Table 4).

### 3.2. Findings from the Qualitative Components

#### 3.2.1. Perspectives on the Current Malaria Situation 

Most respondents were aware of the severity of malaria in the past and persistent malaria transmission in their locations especially during rainy seasons. Some had lost family members most likely due to malaria. Many also reported that contracting malaria could lead to economic or financial burden to the family due to taking care of each other without working when one person contracted malaria. Although malaria diagnostic and treatment services are freely available in their villages, respondents received malaria-related health information only occasionally.

“In my family, my son passed away due to malaria in the last five years when malaria was very common in this village. Nowadays, we can easily receive a diagnosis of malaria and quality antimalarial drugs when malaria is detected. Still, many malaria cases are reported especially during rainy season.”

(IDI, 35-year-old man)

“Malaria was very serious in the old days. When one family member contracted malaria, other people had to take care of the patient which could lead to reduced family income. To date, malaria remains a health problem in our village, and we are taking good care to prevent it.”

(IDI, 28-year-old woman)

“In my family, everyone contracted malaria two to three times already. Healthcare providers and village malaria volunteers are always ready to provide diagnostic and treatment services. Nonetheless, we still want more malaria-related health information whenever possible.”

(IDI, 31-year-old man)

#### 3.2.2. Challenges Faced by Respondents Adhering to Malaria Preventive Measures Including LLIN Use

Most respondents described biting mosquitos as the source of malaria transmission. However, they did not always use LLINs due to several reasons. To prevent malaria, female respondents always used conventional nets, whereas males rarely used any nets due to the nature of their work. Still, as a misconception, many respondents avoided eating several fruits, e.g., banana, and drinking stagnant water as malaria preventive measures. Many female respondents revealed that newly received LLINs were usually kept for future use by visitors if any.

“I am aware that malaria is transmitted by biting mosquitos. Therefore, I always use bed nets either conventional or insecticidal nets when I am sleeping. Moreover, I also avoid eating bananas and drinking stream water when travelling in the forest.”

(IDI, 27-year-old man)

“In our family, we have several insecticidal nets distributed by malaria officers. Nevertheless, I am a gold miner working in the forest especially during night-time, so using LLINs is not possible. Therefore, I just wear long-sleeved clothes to prevent biting of mosquitos and other insects.”

(IDI, 35-year-old man)

“In my family, we have used LLINs especially during nighttime. As for me, I prefer to use conventional ones which use a thick layer nylon net. Still, we have some new LLINs which are kept for visitors if any.”

(IDI, 35-year-old woman)

#### 3.2.3. Perceived Possible Solutions to Overcome the Obstacles towards the Prevention of Malaria

Almost all respondents wanted regular health education sessions by healthcare providers regarding the proper use of LLINs following the frequent distribution of LLINs. Male respondents suggested receiving alternative personal protective equipment rather than LLINs to use in forest-related settings where LLINs are not feasible to set up. Some asked healthcare providers to distribute mosquito repellents free of charge. As such, they can prevent mosquito bites especially for their children before getting under mosquito nets before sleeping at night.

“I am willing to use LLIN whenever and wherever I am sleeping. Nonetheless, as I work and reside in the farm, I could not use any net. However, I burned rubbish or used mosquito coils to prevent mosquito bites. If possible, I do want any kind of mosquito repellent provided by the malaria project.”

(IDI, 29-year-old man)

“We usually use mosquito nets or LLINs at night when many mosquitoes emerged. Still, we burn mosquito coils to prevent mosquito bites before sleeping. Therefore, it would be useful if we could receive mosquito repellents especially for young children.”

(IDI, 42-year-old woman)

## 4. Discussion

In this study, people residing in moderate- and high-malaria-endemic villages revealed more poor malaria preventive practices than those of low-endemic villages. Many studies in Myanmar also documented poor malaria preventive practice behaviors among people from malarious areas [16,20,22,23]. More resource allocations should be delivered for conducting more awareness-raising activities, especially in high-endemic villages. Given that most respondents possessed misconceptions towards the reasons for malaria transmission, awareness-raising activities should focus on eradicating misconceptions. As a historic belief, many people from Myanmar still perceived that eating banana and papaya and drinking stagnant water were causative agents of malaria [16,24]. The delivery of health education by respectful individuals such as Buddhist monks and village leaders should be considered for effectively disseminating error-free messages. Using mass media announcements that disseminate simple and short health messages might also be effective [25].

In Myanmar, everyone over five years of age should enter school to start their education journey. Once they are 18 years old, whether they have obtained a bachelor’s degree or not, adults should be responsible for the family business, especially in rural areas. Therefore, adults often access outreach health care activities. Sometimes, they cannot even follow conventional malaria preventive practices due to the nature of their jobs, e.g., fishing in the sea or gold mining at night-time. One study in Myanmar also corroborated the finding that adults usually maintain the poorest rate of LLIN use compared to children and women [26]. Night-time health talks for those returning from work in the evening should be considered. Sharing health knowledge should also be strengthened by other family members who have participated in health education campaigns.

Myanmar is said to be a patriarchal country in which males are often family heads. Nevertheless, males usually showed more poor malaria preventive practices than females [27]. One study in eastern Myanmar also states that females use insecticidal nets more than males [26]. Therefore, male-oriented health promotion activities should be introduced. Next, the current health interventions are targeted to the general population in which females are usually involved [16]. Participation by male groups should be promoted. Moreover, female participants should increase their awareness to disseminate the knowledge gained from community health education activities back to their partners or other family members. Likewise, males from rural townships generally work as farmers or gold miners to seek income. Because of the nature of their work, they sometimes cannot use malaria preventive personal protective equipment, e.g., setting up long-lasting insecticide-treated nets while working in the forest is unreliable. Consequently, malaria preventive practices among forest-related workers in Myanmar are usually low [22,28]. To expand malaria surveillance coverage and to deliver health promotion activities in remote areas, supporting a malaria volunteer workforce is one of the effective options [29]. Therefore, recruiting worksite malaria volunteers to implement malaria control activities including health-awareness-raising campaigns may solve this problem. Perhaps new malaria preventive equipment harmonized for use in forest settings should be created, e.g., insecticide-treated clothes.

In Myanmar, malaria-related commodities, including LLINs, are distributed free of charge by the government and other implementing malaria partners to the community. Further, people can receive free malaria diagnostic and treatment services at the nearest health centers and from trained village malaria volunteers [28]. Still, people from rural families are busy struggling to overcome financial hardship amid the ongoing COVID-19 pandemic and emerging political affairs in Myanmar. Subsequently, people possessing low annual family incomes likewise exhibited poor malaria preventive practices. The result is supported by related research in Laos where respondents with low family incomes exhibited a greater chance of displaying poor malaria preventive practices [27]. Social capital development projects should also be expanded in rural areas together with intensive disease control activities. 

The qualitative findings suggested that people were usually excluded from malaria prevention, mostly due to work-related activities despite expressing a willingness to use distributed LLINs. The use of LLINs in this group of individuals (farmers, gold miners, etc.) is usually low, as stated by studies in central Vietnam [30] and Myanmar [31]. When the possession of LLINs is high, the use of LLINs should be promoted in the community. The use of personal protective measures for those who are unable to use LLINs should be strongly promoted. Collaboration with private gold mining companies to deliver health promotion activities among gold miners should be implemented. Distributing malaria protection kits including mosquito repellents and other insect-repellent-emulsion-coated materials to forest travelers should be initiated. 

This study contained some caveats. First, we found differences in malaria preventive practices among people residing in villages with different malaria endemicities. The statistical tests constructing a direct link between malaria preventive practices and the persistent malaria burden remain lacking. Nevertheless, using the reported malaria data in each village and detailed sampling procedures encountered upon choosing study villages would tend to produce representative results. Second, the entomologic conditions (malaria vector bionomics) [32] and environmental indicators might differ among each village. Nonetheless, we selected villages from a single cluster, i.e., the same village tract, in a township. Therefore, people residing in this area might experience similar risks of contracting malaria, similar volumes of population displacements, and equal malaria vector distribution. Another comprehensive study including the analysis of entomologic and environmental predictors is recommended.

## 5. Conclusions

This study documented the disparities of malaria preventive practices among community members within the village tracts with different endemic settings. The results also pointed out the needs of village-based micro-stratification plans, including detailed resource allocation. Health education activities related to malaria preventive practices should be increased especially in moderate- and high-malaria-endemic villages. Targeted groups should include adult male farmers or gold miners from families with low annual incomes. Moreover, promoting the use of insecticidal nets and creating new personal protective equipment that is reliable for use in forest settings are encouraged. Specific interventions to eliminate misconceptions about malaria causation and prevention should also be introduced. For those who cannot use LLINs during their working time, the use of personal protective measures such as wearing long-sleeved clothing and applying mosquito repellents should be promoted.

## Figures and Tables

**Table 1:** General characteristics of respondents in each malaria-endemic setting.

Characteristic	Low-Endemic (n = 120)	Moderate-Endemic (n = 120)	High-Endemic (n = 120)	*p*-Value
	n (%)	n (%)	n (%)	
Age (years)				0.833
18–30	35 (29.2)	40 (33.4)	42 (35.0)	
>30–45	50 (41.6)	43 (35.8)	43 (35.8)	
>45	35 (29.2)	37 (30.8)	35 (29.2)	
Gender				0.620
Female	64 (53.3)	65 (54.2)	58 (48.3)	
Male	56 (46.7)	55 (45.8)	62 (51.7)	
Occupation				0.773
Unemployed	21 (17.5)	17 (14.2)	24 (20.0)	
Farmers	56 (46.7)	52 (43.3)	56 (46.6)	
Gold miners	32 (26.7)	38 (31.7)	32 (26.7)	
Others	11 (9.1)	13 (10.8)	8 (6.7)	
Education				0.769
Illiterate	18 (15.0)	20 (16.7)	20 (16.7)	
Primary school	56 (46.7)	67 (55.8)	61 (50.8)	
Middle school	34 (28.3)	25 (20.8)	29 (24.2)	
High school and above	12 (10.0)	8 (6.7)	10 (8.3)	
Family members				0.314
<3	22 (18.3)	18 (15.0)	15 (12.5)	
3–5	45 (37.5)	51 (42.5)	61 (50.8)	
>5	53 (44.2)	51 (42.5)	44 (36.7)	
Annual family income (MMK) ^a^				0.372
<1,000,000	36 (30.0)	44 (36.7)	31 (25.8)	
1,000,000–2,000,000	61 (50.8)	58 (48.3)	70 (58.4)	
>2,000,000	23 (19.2)	18 (15.0)	19 (15.8)	

^a^ 1 USD ~2100 MMK; low-endemic: annual parasite incidence (API) < 1; moderate-endemic: API = 1 to 5; high-endemic: API > 5; *p*-value by chi-squared test; degree of freedom = 1.

**Table 2:** Knowledge on causation of malaria and access to malaria-related health information.

Statement	Low-Endemic (n = 120)	Moderate-Endemic (n = 120)	High-Endemic (n = 120)	*p*-Value
	Yes			
	n (%)	n (%)	n (%)	
What is the causation of malaria?				
Biting of mosquito carrying infected malaria parasites	100 (83.3)	89 (74.2)	91 (75.8)	0.191
Eating fruits such as banana, papaya or other fruits ^a^	11 (9.2)	31 (25.8)	23 (19.2)	0.003 *
Drinking stagnant water ^a^	42 (35.0)	52 (43.3)	61 (50.8)	0.046 *
Living or sleeping in the forest ^a^	31 (25.8)	41 (34.2)	43 (35.8)	0.205
Relapse of malaria due to latent parasites in the human body	20 (16.7)	21 (17.5)	23 (19.2)	0.875
Access to malaria-related health information				
Have you ever received malaria-related health information?	82 (68.3)	76 (63.3)	89 (74.2)	0.194
What is the source of information?				0.202
Health care providers (government basic health staff)	62 (75.6)	71 (93.4)	80 (89.9)	
Village malaria volunteers	58 (70.7)	58 (76.3)	67 (75.3)	
Other stakeholders/ village authorities	4 (4.9)	7 (9.2)	16 (18.0)	
How many times have you received it?				0.624
1–2	46 (56.1)	45 (59.2)	52 (58.4)	
3–4	32 (39.0)	23 (30.3)	30 (33.7)	
≥5	4 (4.9)	8 (10.5)	7 (7.9)	
Have you ever received malaria preventive practice-related information?	61 (50.8)	68 (56.7)	72 (60.0)	0.351
What is the source of information?				0.351
Health care providers (Government basic health staff)	58 (95.1)	58 (85.3)	68 (94.4)	
Village malaria volunteers	34 (55.7)	24 (35.3)	30 (41.7)	
Other stakeholders/ village authorities	1 (1.6)	5 (7.4)	5 (6.9)	
How many times have you ever received it?				0.630
1–2	45 (73.8)	52 (76.5)	50 (69.4)	
3–4	13 (21.3)	10 (14.7)	14 (19.4)	
≥5	3 (4.9)	6 (8.8)	8 (11.1)	

^a^ Negative questions or statements; low-endemic: annual parasite incidence (API) < 1; moderate-endemic: API = 1 to 5; high-endemic: API > 5; *p*-value by chi-squared test; * statistical significance at *p* < 0.05; degree of freedom = 1.

**Table 3:** Malaria preventive practices among respondents from each malaria-endemic setting.

Statement	Low-Endemic (n = 120)	Moderate-Endemic (n = 120)	High-Endemic (n = 120)
	Yes		
	n (%)	n (%)	n (%)
What malaria preventive practices (methods) are you currently following?			
Use of long-lasting insecticide-treated nets	105 (87.5)	76 (63.3)	67 (55.8)
Use of conventional bed nets	15 (12.5)	34 (28.3)	41 (34.2)
Avoid eating banana, papaya or other fruits ^a^	13 (10.8)	12 (10.0)	18 (15.0)
Avoid drinking stagnant water ^a^	67 (55.8)	80 (66.7)	76 (63.3)
Personal protective measures (wearing long-sleeved shirts or pants, applying mosquito repellents)	32 (26.7)	41 (34.2)	41 (34.2)
Burning rubbish or mosquito coils ^a^	51 (42.5)	68 (56.7)	70 (58.3)
Practice regarding long-lasting insecticide-treated nets (LLINs)			
Did you sleep under LLINs last night before the survey?	104 (86.7)	71 (59.2)	60 (50.0)
Is an average of one LLIN per two individuals sufficient for your family?	118 (98.3)	114 (95.0)	117 (97.5)
Did you receive LLINs within the last two years?	117 (97.5)	111 (92.5)	116 (96.7)
Have you already washed this current using LLINs more than 20 times? ^a^	22 (18.3)	31 (25.8)	40 (33.3)

^a^ Negative questions or statements.

**Table 4:** Factors associated with poor malaria preventive practices among people residing in moderate- and high-malaria-endemic villages.

Characteristic	Moderate-Endemic (n = 120)				High-Endemic (n = 120)			
	Poor Practice n (%)	Good Practice n (%)	cOR (95%CI)	aOR (95%CI)	Poor Practice n (%)	Good Practice n (%)	cOR (95%CI)	aOR (95%CI)
Age (years)								
18–30	18 (45.0)	22 (55.0)	3.0 (1.2–8.1)	3.1 (1.3–8.6)	24 (57.1)	18 (42.9)	2.9 (1.1–7.4)	2.9 (1.4–8.1)
>30–45	20 (46.5)	23 (53.5)	3.2 (1.2–8.5)	3.4 (1.5–9.1)	24 (55.8)	19 (44.2)	2.8 (1.1–7.0)	2.8 (1.6–11.0)
>45	8 (21.6)	29 (78.4)	Ref.	Ref.	11 (31.4)	24 (68.6)	Ref.	Ref.
Gender								
Female	18 (27.7)	47 (72.3)	Ref.	Ref.	20 (34.5)	38 (65.5)	Ref.	Ref.
Male	28 (50.9)	27 (49.1)	2.7 (1.3–5.8)	3.1 (2.0–8.2)	39 (62.9)	23 (37.1)	3.2 (1.5–6.8)	3.6 (2.0–9.7)
Occupation								
Unemployed	3 (17.6)	14 (82.4)	Ref.	Ref.	7 (29.2)	17 (70.8)	Ref.	Ref.
Farmers	24 (46.2)	28 (53.8)	4.0 (1.0–15.6)	4.1 (1.0–16.1)	32 (57.1)	24 (42.9)	3.2 (1.2–9.0)	3.5 (1.9–11.2)
Gold miners	18 (47.4)	20 (52.6)	4.2 (1.0–17.0)	4.2 (1.5–19.5)	17 (53.1)	15 (46.9)	2.8 (1.0–8.4)	2.9 (1.6–10.0)
Others	1 (7.7)	12 (92.3)	0.4 (0.0–4.3)	0.4 (0.1–4.4)	3 (37.5)	5 (62.5)	1.5 (0.3–7.8)	1.4 (0.2–6.7)
Education								
Illiterate	9 (45.0)	11 (55.0)	5.7 (0.6–55.6)	6.1 (0.9–57.6)	9 (45.0)	11 (55.0)	1.9 (0.4–9.6)	2.0 (0.8–11.2)
Primary school	30 (44.8)	37 (55.2)	5.7 (0.7–48.7)	5.5 (0.2–44.3)	38 (62.3)	23 (37.7)	3.9 (0.9–16.4)	3.7 (0.8–15.6)
Middle school	6 (24.0)	19 (76.0)	2.2 (0.2–21.8)	2.2 (0.3–20.8)	9 (31.0)	20 (69.0)	1.1 (0.2–5.0)	1.1 (0.1–4.3)
High school and above	1 (12.5)	7 (87.5)	Ref.	Ref.	3 (30.0)	7 (70.0)	Ref.	Ref.
Family members								
<3	6 (33.3)	12 (66.7)	Ref.	Ref.	7 (46.7)	8 (53.5)	Ref.	Ref.
3–5	19 (37.3)	32 (62.7)	1.2 (0.4–3.7)	1.5 (0.8–4.1)	31 (50.8)	30 (49.2)	1.2 (0.4–3.7)	1.2 (0.3–3.1)
>5	21 (41.2)	30 (58.8)	1.4 (0.5–4.3)	1.4 (0.4–3.9)	21 (47.7)	23 (52.3)	1.0 (0.3–3.4)	0.8 (0.1–2.1)
Annual family income (MMK) ^a^								
<1,000,000	20 (45.5)	24 (54.5)	4.2 (1.1–16.5)	4.7 (1.7–20.2)	18 (58.1)	13 (41.9)	3.9 (1.1–13.5)	5.0 (2.0–17.5)
1,000,000–2,000,000	23 (39.7)	35 (60.3)	3.3 (0.9–12.6)	4.1 (1.6–14.7)	36 (51.4)	34 (48.6)	3.0 (0.9–9.2)	3.9 (2.1–11.3)
>2,000,000	3 (16.7)	15 (83.3)	Ref.	Ref.	5 (26.3)	14 (73.7)	Ref.	Ref.

^a^ 1 USD ~2100 MMK; low-endemic: annual parasite incidence (API) < 1; moderate-endemic: API = 1 to 5; high-endemic: API > 5; *p*-value by chi-squared test; cOR: crude odds ratio; aOR: adjusted odds ratio; 95%CI: 95% confidence interval; Ref.: reference.

## Data Availability

All data of this research are included in the manuscript.

## Supplementary Materials

The following supporting information can be downloaded at: https://www.mdpi.com/article/10.3390/tropicalmed7110353/s1, Table S1: Factors associated with poor malaria preventive practices among people residing in low-malaria-endemic villages.
