# Supplementary material for: Malaria Preventive Practices among People Residing in Different Malaria-Endemic Settings in a Township of Myanmar: A Mixed-Methods Study
Source: Trop Med Infect Dis. 2022 Nov 4;7(11):353. doi: 10.3390/tropicalmed7110353 (PMC9692550; doi:10.3390/tropicalmed7110353)
Supplement: Supplementary file 1 [file tropicalmed-07-00353-s001.zip › tropicalmed-1961242-supplementary.pdf]

Table S1. Factors associated with poor malaria preventive practices among people residing in low-malaria-endemic villages.

Characteristics	Low endemic (n = 120)			
	Poor practice n (%)	Good practice n (%)	cOR (95%CI)	aOR (95%CI)
Age (years)				
18–30	28 (80.0)	7 (20.0)	0.5 (0.1–2.0)	0.6 (0.2–3.8)
>30–45	44 (88.0)	6 (12.0)	0.9 (0.2–3.6)	1.2 (0.5–5.4)
>45	31 (88.6)	4 (11.4)	Ref.	Ref.
Gender				
Female	53 (82.8)	11 (17.2)	Ref.	Ref.
Male	50 (89.3)	6 (10.7)	1.7 (0.6–5.0)	2.1 (0.9–7.2)
Occupation				
Unemployed	18 (85.7)	3 (14.3)	Ref.	Ref.
Farmers	49 (87.5)	7 (12.5)	1.2 (0.3–5.0)	1.1 (0.6–6.5)
Goldminers	27 (84.4)	5 (15.6)	0.9 (0.2–4.2)	0.9 (0.4–5.1)
Others	9 (81.8)	2 (18.2)	0.8 (0.1–5.3)	1.0 (0.6–8.1)
Education				
Illiterate	13 (72.2)	5 (27.8)	0.2 (0.0–2.3)	0.2 (0.1–3.2)
Primary school	48 (85.7)	8 (14.3)	0.5 (0.1–4.8)	0.8 (0.6–7.2)
Middle school	31 (91.2)	3 (8.8)	0.9 (0.1–10.0)	1.7 (0.3–15.3)
High school and above	11 (91.7)	1 (8.3)	Ref.	Ref.
Family members				
<3	19 (86.4)	3 (13.6)	Ref.	Ref.
3–5	38 (84.4)	7 (15.6)	0.9 (0.2–3.7)	1.1 (0.5–4.6)
>5	46 (86.8)	7 (13.2)	1.0 (0.2–4.4)	1.4 (0.6–7.1)
Annual family income (MMK) [a]				
<1,000,000	30 (83.3)	6 (16.7)	0.5 (0.1–2.6)	0.9 (0.4–4.2)
1,000,000–2,000,000	52 (85.2)	9 (14.8)	0.6 (0.1–2.8)	0.4 (0.3–2.9)
>2,000,000	21 (91.3)	2 (8.7)	Ref.	Ref.

[a] 1 USD ~2100 MMK; Low endemic: annual parasite incidence (API) < 1; Moderate endemic: API = 1 to 5; High endemic: API > 5; *p*-value by chi-squared test; cOR: crude odds ratio; aOR: adjusted odds ratio; 95%CI: 95% confidence interval; Ref.: reference.
